# Adaptive Dual Aggregation Network with Normalizing Flows for Low-Light Image Enhancement

**DOI:** 10.3390/e26030184

**Published:** 2024-02-22

**Authors:** Hua Wang, Jianzhong Cao, Jijiang Huang

**Affiliations:** 1 Xi’an Institute of Optics and Precision Mechanics, Chinese Academy of Sciences, Xi’an 710119, China; cjz@opt.ac.cn (J.C.); huangjijiang@opt.ac.cn (J.H.); 2 University of Chinese Academy of Sciences, Beijing 100049, China

**Keywords:** low-light image enhancement, normalizing flow, adaptive dual aggregation, deep learning

## Abstract

Low-light image enhancement (LLIE) aims to improve the visual quality of images taken under complex low-light conditions. Recent works focus on carefully designing Retinex-based methods or end-to-end networks based on deep learning for LLIE. However, these works usually utilize pixel-level error functions to optimize models and have difficulty effectively modeling the real visual errors between the enhanced images and the normally exposed images. In this paper, we propose an adaptive dual aggregation network with normalizing flows (ADANF) for LLIE. First, an adaptive dual aggregation encoder is built to fully explore the global properties and local details of the low-light images for extracting illumination-robust features. Next, a reversible normalizing flow decoder is utilized to model real visual errors between enhanced and normally exposed images by mapping images into underlying data distributions. Finally, to further improve the quality of the enhanced images, a gated multi-scale information transmitting module is leveraged to introduce the multi-scale information from the adaptive dual aggregation encoder into the normalizing flow decoder. Extensive experiments on paired and unpaired datasets have verified the effectiveness of the proposed ADANF.

## 1. Introduction

Insufficient light in complex imaging environments can lead to dark brightness, low contrast, high noise, and poor details in captured images [1,2]. Low-light image enhancement (LLIE) aims to solve the problems of insufficient visibility and low contrast in low-light images while restoring noise, structures, color distortion, etc. [3]. Low-light image enhancement can effectively improve the performance of methods such as object detection and scene understanding at night or in low-light conditions [4].

Over the past decades, many low-light image enhancement methods have been proposed [5,6]. Previous methods are usually based on hand-designed features and processing steps such as histogram equalization [7,8] and gamma transformation [9]. These methods are simple and fast, but they usually amplify noise while enhancing the image and often cannot restore the color and details of low-light images well [10]. The widely popular Retinex theory [11] provides an intuitive and easy-to-understand framework for LLIE by decomposing the image into reflection and illumination components [12,13]. However, for complex illumination properties in practice, it is challenging to design priors and regularizations that are always valid for accurate decomposition of the reflection and illumination components [14,15]. Improper decomposition can lead to unrealistic details, undesirable artifacts, and color distortion in enhanced images [16].

Inspired by the successful application of deep learning in object recognition, detection, etc. [17,18], researchers have focused on building various deep learning frameworks suitable for the LLIE task [6,19,20]. In addition, the development of paired datasets [21,22] has indeed been a critical step in enabling the application of deep learning to the LLIE task. Recent IILE methods based on deep learning can be roughly divided into deep-Retinex-based methods and end-to-end methods [5,23,24].

Deep-Retinex-based methods are also based on the human visual system’s Retinex theory and use neural networks to simulate the process of separating the reflectance and illumination components [25]. These methods aim to combine the advantages of both Retinex theory and deep learning, enabling an interpretable low-light image enhancement paradigm [26,27]. Under low-light conditions, the boundary between the reflectance component and the illuminance component can become blurred, making it more difficult to accurately separate them. Even when using deep learning models for Retinex decomposition, there is still the problem of being unable to accurately separate the reflectance and illumination components, which may lead to noise amplification and image stylization in the enhanced results [28]. In order to solve these problems, researchers are constantly exploring ways to improve deep learning models, such as by using more complex network structures or introducing regularization techniques to improve model performance [10,29].

End-to-end methods typically use deep neural networks to directly learn the non-linear relationships between low-light images and their corresponding normally exposed images [30,31]. By removing the need for explicit separation of the reflectance and illumination components, end-to-end methods focus on designing a variety of novel neural network structures for LLIE [32]. End-to-end methods have the advantage of being less dependent on physical models and can directly learn the desired mapping between low-light and normal-light images [33]. However, they may not be as interpretable as deep-Retinex-based methods due to their black-box nature.

Recent methods based on deep learning have made good progress in LLIE. However, these methods generally use pixel-level error functions, such as L1 or L2 norm, as the objective function of deep networks for training [5,10]. Pixel-level error functions cannot measure the real visual errors between enhanced images and normally exposed images such as complex structures and textures [34,35]. And pixel-level error functions have difficulty providing effective regularization for local structures in various complex backgrounds.

To alleviate the above problem, we propose an adaptive dual aggregation network with normalizing flows (ADANF) for low-light image enhancement. Different from previous methods that use pixel-level error functions to measure the difference between enhanced and normally exposed images in the image domain, we adopt a normalizing flow framework to map enhanced and normally exposed images to the underlying data distribution, which can effectively express the structural details of complex images [36]. In addition, we use the errors between the data distributions for enhanced and normally exposed images as the objective function to effectively measure the visual distance.

In the proposed ADANF, an adaptive dual aggregation encoder is firstly exploited to extract illumination-robust features by fully exploring the global properties and local details of the low-light images. Next, a reversible normalizing flow decoder is leveraged to recover normally exposed images from the illumination-robust features. Here, we exploit the inverse process capabilities of the normalized stream decoder to reconstruct brighter, more detailed images. Finally, to further improve the quality of image enhancement, a gated multi-scale information transmitting module is designed to introduce the multi-scale features from the adaptive dual aggregation encoder into the normalizing flow decoder. Extensive experiments on paired and unpaired datasets verify the effectiveness of the proposed ADANF.

The contributions of this paper mainly include:

- An adaptive dual aggregation encoder is leveraged to fully capture the global properties and local details of low-light images for extracting illumination-robust features from low-light images.
- To measure real visual errors between enhanced and normally exposed images, a reversible normalizing flow decoder is used to map enhanced and normally exposed images to potential distributions, and the difference between the distributions is used as the objective function for training.
- A gated multi-scale information transmitting module is designed to introduce the multi-scale features from the adaptive dual aggregation encoder into the normalizing flow decoder to further improve the quality of enhanced images.

The rest of the manuscript is organized as follows. Recent related works are introduced in Section 2. Section 3 gives the details of the proposed ADANF. Section 4 reports experimental results. Finally, the conclusion is provided in Section 5.

## 2. Related Work

### 2.1. Traditional Methods

Previous methods usually study hand-designed features for LLIE. Histogram equalization is one of the most classic low-light image enhancement methods [37]. Reza [38] designed a block-based histogram equalization method to model lighting changes in local areas. Lee et al. [39] calculated the 2D histogram by considering the relationship between neighboring pixels within local regions. They utilized the layered difference approach for enhancing contrast. In addition, some researchers attempted to combine image quality assessment with histogram equalization to improve performance. Gu et al. [40] used subjective and objective evaluation guidance to improve the histogram to correct image brightness and contrast to the level of normal exposure.

Retinex theory is also very popular in low-light image enhancement, and researchers have carefully designed many decomposition methods based on the Retinex theory. Kimmel et al. [41] proposed to introduce the lighting component gradient into a variational framework for LLIE. Ren et al. [12] designed a low-rank prior regularized Retinex decomposition model to alleviate the noise amplification problem. Gu et al. [13] proposed a fractional-order variational structure that regularizes both the reflectance and illumination components. Liang et al. [42] combined nonlinear diffusion techniques and Retinex decomposition to estimate lighting components to improve estimation results. These methods are sensitive to illumination changes. In low-light environments, illumination changes may lead to inaccurate feature extraction and affect the enhancement effect.

### 2.2. Deep-Learning-Based Methods

Recent methods mainly study the design of deep learning frameworks for LLIE, including deep-Retinex-based methods and end-to-end methods [5,10]. Deep-Retinex-based methods combine the advantages of Retinex theory and deep learning to provide an interpretable solution for low-light image enhancement. Wei et al. [21] proposed a Retinex-Net including Decom-Net and Enhance-Net. Decom-Net is responsible for decomposing the input low-light image into reflection and illumination parts, while Enhance-Net is responsible for enhancing the illumination part to obtain normally exposed images. Zhang et al. [43] proposed a KinD network to utilize images under different exposure conditions for training. Fan et al. [29] introduced a semantic segmentation sub-network into the Retinex model to use semantic priors to guide image enhancement. Liu et al. [27] employed unrolling and adjustment to exploit global and local brightness of images for LLIE.

End-to-end methods focus on carefully designing different networks to learn the mapping between low-light and normally exposed images. Lore et al. [20] designed the first deep network LLNet for LLIE, which is a sparse denoising autoencoder structure. Yang et al. [33] proposed to exploit a transformer-based network to extract the global information of low-light images. Ren et al. [44] utilized an encoder–decoder network to extract global content and a recurrent neural network to preserve edge details. Xu et al. [45] proposed a frequency-based model that uses low-frequency layers to restore content and high-frequency layers to restore image details. Xu et al. [31] considered that the information amounts in different areas are different and designed a signal-to-noise-ratio-aware transformer for LLIE. However, recent deep-learning-based methods usually employ pixel-level L1 or L2 norm as the objective function to optimize deep networks, which cannot effectively measure the real visual errors between the enhanced image and the normal exposure image.

## 3. Methods

LLIE aims at generating the normally exposed image $X_{n}\in R^{H\times W\times 3}$ from a low-light image $X_{l}\in R^{H\times W\times 3}$, where *W* and *H* represent the width and height, respectively. Previous methods focus on studying different networks, directly utilizing MSE [20], L1 [46], or color loss [47] as objective functions to perform supervised training under paired training samples $\left\{X_{l},X_{gt}\right\}$, where $X_{gt}\in R^{H\times W\times 3}$ is the ground truth normally exposed image. However, there are two problems with previous methods. First, it is difficult for these methods to fully adaptively utilize the global and local information of the image $X_{l}$ to improve visual effect and suppress noise. On the other hand, the loss functions of these methods focus on pixel level or local errors, and it is difficult to fully utilize the visual properties to measure the real visual errors between the generated image $X_{n}$ and the ground truth $X_{gt}$ [35].

**Figure 1:**
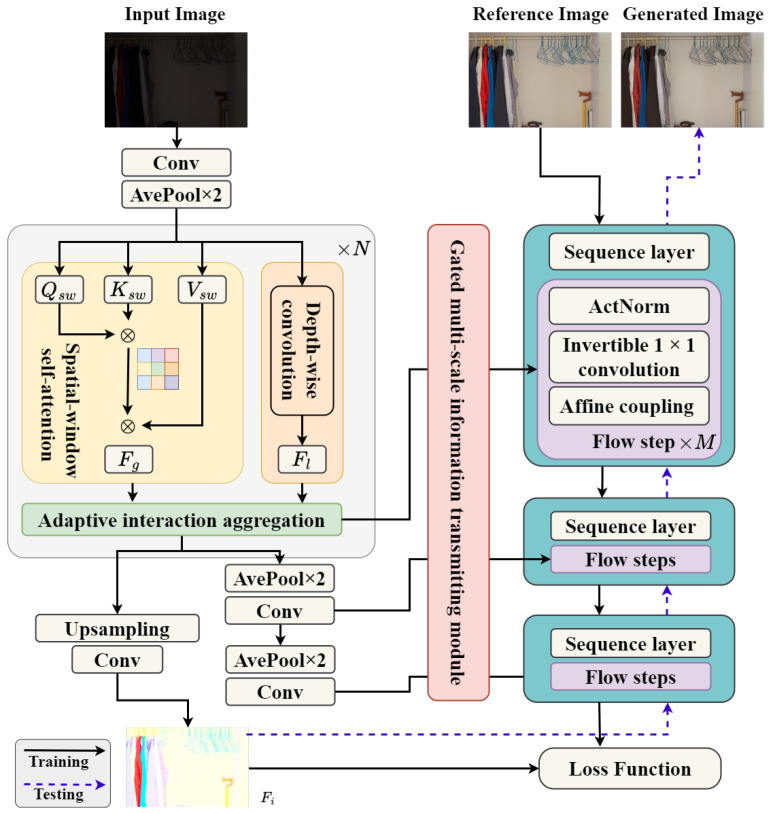
Detailed structures of ADANF. In the testing phase, a low-light image is first fed to the adaptive dual aggregation encoder to fully exploit the global properties and local details for extracting illumination-robust features. Then, a gated multi-scale information transmitting module is designed to introduce the multi-scale features of the adaptive dual aggregation encoder into the normalizing flow decoder. Finally, an invertible normalizing flow decoder is used to recover the normally exposed image from the illumination-robust features.

To alleviate the above two problems, an adaptive dual aggregation network with normalizing flows (ADANF) is proposed for LLIE. The overall structures of the ADANF are shown in Figure 1. First, an adaptive dual aggregation encoder is employed to fully exploit the global properties and local details of the image $X_{l}$ to extract illumination-robust features. Then, an invertible normalizing flow decoder is used to recover the normally exposed image $X_{n}$ from the illumination-robust features. Finally, a gated multi-scale information transmitting module is designed to introduce the multi-scale features of the adaptive dual aggregation encoder into the normalizing flow decoder to further improve the quality of image enhancement.

### 3.1. Adaptive Dual Aggregation Encoder

#### 3.1.1. Preprocessing

Low-light images often have local or global dark areas, resulting in poor contrast and unclear detail. In addition, insufficient light may also cause problems such as noise and artifacts. If the original low-light images are input directly into the model, the model may have difficulty distinguishing low-contrast areas and noisy areas. By performing histogram equalization on $X_{l}$, we can redistribute the pixel intensities of an image $X_{l}$ so that they occupy the entire possible intensity range. The histogram-equalized image $h\left(X_{l}\right)\in R^{H\times W\times 3}$ will have higher contrast and the model can more easily identify and perceive different areas in the image. In addition, we use color map $c\left(X_{l}\right)\in R^{H\times W\times 3}$ to enhance the contrast and visibility of low-light images $X_{l}$, highlighting details in dark areas, where $c\left(X_{l}\right)=X_{l}/{\mathrm{mean}}_{p}\left(X_{l}\right)$, and ${\mathrm{mean}}_{p}\left(X_{l}\right)$ represents the calculation of the mean value of each pixel in $X_{l}$. Finally, we use the gradient map $g\left(X_{l}\right)\in R^{H\times W\times 3}$ to explicitly capture the noisy areas in low-light images $X_{l}$, where $g\left(X_{l}\right)=\max\left(\left|\nabla_{x}\left(c\left(X_{l}\right)\right)\right|,\left|\nabla_{y}\left(c\left(X_{l}\right)\right)\right|\right)$, $\nabla_{x}$, and $\nabla_{y}$ are the gradients in the *x* and *y* directions, respectively. To improve the model’s sensitivity to noisy areas in low-contrast and dark areas, $h(X_{l})$, $c(X_{l})$, $g(X_{l})$, and $X_{l}$ are stacked by channel as the input $X_{in}=\left[h\left(X_{l}\right),c\left(X_{l}\right),g\left(X_{l}\right),X_{l}\right]$ of the subsequent network.

#### 3.1.2. Global–Local Adaptive Aggregation Module

In the adaptive dual aggregation encoder, two 3×3 convolutions are first used to transform the image $X_{in}$ into the feature space to obtain the shallow feature $F_{s}\in R^{H\times W\times C_{s}}$, where $C_{s}$ is the channel number. Then, global–local adaptive aggregation blocks are used to extract illumination-robust feature $F_{i}\in R^{H\times W\times C_{i}}$. The global–local adaptive aggregation block is the key module of the adaptive dual aggregation encoder, and we take a global–local adaptive aggregation block as an example to introduce its details.

First, spatial-window self-attention [48,49] is utilized to explore the global information of the image. We generate query features $Q\in R^{H\times W\times C_{i}}$, key features $K\in R^{H\times W\times C_{i}}$, and value features $V\in R^{H\times W\times C_{i}}$ from the shallow feature $F_{s}$ by using convolutions.

(1) $$Q=W_{Q}F_{i},K=W_{K}F_{i},V=W_{V}F_{i},$$

where $W_{Q},W_{K},W_{V}\in R^{1\times 1\times C_{i}}$ are the weights of a 1×1 convolution, and biases are omitted. Since performing self-attention directly on the global image will introduce a huge amount of calculation, we follow SwinTransformer [50] to perform the spatial-window self-attention to reduce the amount of calculation. *Q*, *K*, and *V* are divided into non-overlapping spatial windows $Q_{sw}^{j}$, $K_{sw}^{j}$, and $V_{sw}^{j}\in R^{H_{sw}\times W_{sw}\times C_{i}}$, respectively. $H_{sw}\times W_{sw}$ is the size of the spatial window. We can calculate the features of each spatial window using Equation (2).

(2) $$F_{g}^{j}=\mathrm{softmax}\left(Q_{sw}^{j}{\left(K_{sw}^{j}\right)}^{T}/\sqrt{C_{i}}+P^{j}\right)V_{sw}^{j},$$

where $P^{j}$ is relative position encoding of the *j*-th spatial window. The outputs of spatial-window self-attention are $F_{g}=\left[F_{g}^{1},F_{g}^{2},\cdot ,F_{g}^{n}\right]$, where $n={\left(H/H_{sw}\right)}^{2}$ is the number of spatial windows and $F_{g}\in R^{H\times W\times C_{i}}$. In addition, shift window operations [50] are utilized to extract the global spatial feature of the image.

Second, to capture details and textures in images of LLIE, a local branch uses depth-wise convolution (DWC) operations to extract local features $F_{l}=\mathrm{DWC}\left(V\right)\in R^{H\times W\times C_{i}}$ from the value features *V* from Equation (1).

Third, to fully utilize the global and local information of the image $X_{l}$ to generate illumination-robust features, an adaptive interaction aggregation (AIA) module is designed. Since $F_{g}$ is the global information of the image and $F_{l}$ is the local features of the image, $F_{g}$ and $F_{l}$ are misaligned features. In this case, simple feature weighted combination or concatenation operations cannot fully integrate global and local information. In the AIA module, we first use the information of local features $F_{l}$ to refine the texture detail information of global features $F_{g}$ by exploiting the attention mechanism. The spatial attention map $S\left(F_{l}\right)\in R^{H\times W\times 1}$ of the local features is calculated as

(3) $$S\left(F_{l}\right)=\varphi \left(W_{sa2}\sigma \left(W_{sa1}F_{l}\right)\right),$$

where φ is the sigmoid activation, σ is the RELU activation, $W_{sa1},W_{sa2}\in R^{1\times 1\times C_{i}}$ are weights of the 1×1 convolutions, $W_{sa1}$ contains $C_{i}$ kernels, and $W_{sa2}$ contains a kernel. Then, we can obtain the refined global feature ${\hat{F}}_{g}=F_{g}⊙S\left(F_{l}\right)$, where ⊙ is the Hadamard product, ${\hat{F}}_{g}\in R^{H\times W\times C_{i}}$. Then, the AIA module utilizes the rich channel information of global features $F_{g}$ to suppress redundant channels of local features $F_{l}$. The channel attention map $C\left(F_{g}\right)\in R^{1\times 1\times C_{i}}$ of $F_{g}$ is

(4) $$C\left(F_{g}\right)=\varphi \left(W_{ca2}\sigma \left(W_{ca1}\mathrm{GAP}\left(F_{g}\right)\right)\right),$$

Then, we can obtain the refined local feature ${\hat{F}}_{l}=F_{l}⊙C\left(F_{g}\right)$, ${\hat{F}}_{l}\in R^{H\times W\times C_{i}}$.

Finally, the refined global and local features ${\hat{F}}_{g}$ and ${\hat{F}}_{l}$ are aggregated by element-wise addition as the output. Multiple global–local adaptive aggregation blocks are repeated to generate illumination-robust feature $F_{i}$.

### 3.2. Normalizing Flow Decoder

During real imaging, changes in lighting conditions (e.g., different time, weather, or light sources) can cause even the same scene to look completely different in low-light images. That is, a normally exposed image will correspond to many different low-light images. A good LLIE method should be able to adapt to changes in lighting conditions. In this paper, we propose to exploit a normalizing flow decoder to recover normally exposed images from illumination-robust feature $F_{i}$.

In the proposed ADANF, the normalizing flow decoder is an invertible network, whose purpose is to learn a one-to-many mapping relationship for LLIE. In the training phase, the normalizing flow decoder aims to learn the mapping of normally exposed images to the feature $F_{i}$ of low-light images [51,52]. The normalizing flow network can adapt to various characteristics of the same scene under different lighting conditions. During the testing phase, the inverse of the learned mapping can be exploited to generate normally exposed images from low-light image features $F_{i}$.

The structures of the normalizing flow decoder has three levels, with a squeeze layer and 12 flow steps at each level. A squeeze layer is a type of layer that reduces the spatial resolution of the input data, which can help with reducing the computational complexity of the network. The flow steps are the main part of the invertible network, where the invertible mapping from normally exposed images to the feature of low-light images is learned.

As shown in Figure 1, a flow step is composed of an activation normalization (ActNorm) layer, an invertible 1×1 convolution, and an affine coupling component. The ActNorm layer is similar to batch normalization, using the scale $\mu \in R^{1\times 1\times C_{i}}$ and bias $\sigma \in R^{1\times 1\times C_{i}}$ parameters of each channel of the input data to perform a transformation $Y_{i}=\frac{F_{i}-\mu }{\sigma }\in R^{H\times W\times C_{i}}$ as preprocessing, whose purpose is to make the input data $F_{i}$ have zero mean and unit variance. The scale μ and bias σ parameters of the ActNorm layer are learnable and initialized using the mean and variance of batch features.

After the ActNorm layer, an invertible 1×1 convolution is used to increase the information interaction between the feature channels of $Y_{i}$ to obtain ${\bar{Y}}_{i}\in R^{H\times W\times C_{i}}$. In invertible 1×1 convolutions, given the output data and convolution kernel, we can accurately recover the original input data. In this way, we can reconstruct the normally exposed image from the feature $F_{i}$ based on the inverse of the learned mapping. In order to make the traditional 1×1 convolution invertible, we need to set its weight matrix to a random orthogonal matrix [51].

The affine coupling component is a special reversible transformation component that can effectively map input data to different feature spaces. It transforms existing channels through multiplication and addition operations and can effectively facilitate the normalizing flow decoder to learn the mapping from normally exposed imgages to the feature $F_{i}$ during the training phase. In the affine coupling component, a split operation is first utilized to divide the input data ${\bar{Y}}_{i}$ into two data, ${\bar{Y}}_{i}^{1}\in R^{H\times W\times C_{i}/2}$ and ${\bar{Y}}_{i}^{2}\in R^{H\times W\times C_{i}/2}$ along the channel dimension. Then, we perform an identity transformation on ${\bar{Y}}_{i}^{1}$ to obtain $H_{1}={\bar{Y}}_{i}^{1}$ and perform an affine transformation on ${\bar{Y}}_{i}^{1}$ to obtain $H_{2}$,

(5) $$H_{2}=\exp\left({\mathrm{NN}}_{s}\left({\bar{Y}}_{i}^{1}\right)\right)⊙{\bar{Y}}_{i}^{2}+{\mathrm{NN}}_{b}\left({\bar{Y}}_{i}^{1}\right),$$

where ${\mathrm{NN}}_{s}\left({\bar{Y}}_{i}^{1}\right)$ and ${\mathrm{NN}}_{b}\left({\bar{Y}}_{i}^{1}\right)$ are shadow three-layer convolutional neural networks to learn the scale and bias from $Y_{i}^{1}$ for affine transformation. Next, $H_{1}$ and $H_{2}$ are concatenated by channel and input into invertible 1×1 convolutions for information interaction among channels. Similar to recent methods [51,52], the flow step is repeated 12 times to learn the mapping.

### 3.3. Mapping Learning Aided by Multi-Scale Features

Due to complex low-light conditions, the detailed information of the image at different scales will be lost or obscured, or the areas at different scales will be too dark or too bright [53]. The multi-scale information of the image is important for LLIE, but the above normalizing flow decoder cannot effectively utilize its multi-scale information. In the proposed ADANF, a gated multi-scale information transmitting module is used to introduce the multi-scale features of the adaptive dual aggregation encoder into the normalizing flow decoder to further improve the quality of image enhancement.

**Figure 2:**
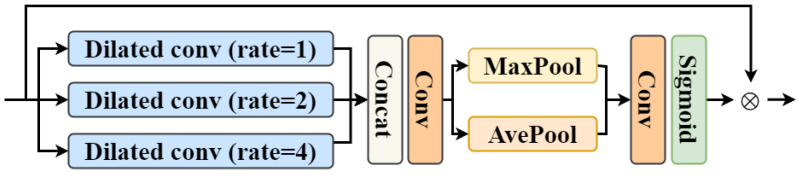
Detailed structures of the gated multi-scale information transmitting module.

Detailed structures of the gated multi-scale information transmitting module are shown in Figure 2. Three dilated convolutions with different dilation rates (e.g., [1,2,4]) are firstly used in parallel to extract the features of different scales [54]. Then, these features are concatenated and fed to 1×1 convolutions to generate multi-scale features $F_{ms}\in R^{H\times W\times C_{i}}$. Next, Global Average-Pooling (GAP) and Global Max-Pooling (GMP) operations in the channel dimension are utilized to extract spatial information $\mathrm{GAP}(F_{ms})$ and $\mathrm{GMP}(F_{ms})$. Convolution operations with sigmoid activation are used to generate attention weights from $F_{ms}$ to control the multi-scale information passed to the normalizing flow decoder. The output of the gated multi-scale information transmitting module is the gated multi-scale features ${\bar{F}}_{ms}\in R^{H\times W\times C_{i}}$,

(6) $${\bar{F}}_{ms}=\sigma \left(\mathrm{Conv}\left([\mathrm{GAP}\left(F_{ms}\right),\mathrm{GMP}\left(F_{ms}\right)]\right)\right)⊙F_{ms}.$$

### 3.4. Loss Function

In the ADANF, we use the normalizing flow decoder to capture the conditional distribution $P_{\mathrm{NFD}}\left(X_{gt}|X_{l},\theta \right)$ of a normally exposed image $X_{gt}$ under its low-light image condition $X_{l}$, where θ represents the parameters of the normalizing flow decoder. Since the normalizing flow decoder is an invertible network, it can map a normally exposed image $X_{gt}$ to a latent variable $z={\mathrm{NFD}}_{\theta }\left(X_{gt};X_{l}\right)$ under the low-light image condition $X_{l}$ and can also reversibly map the latent variable *z* to the normally exposed image $X_{gt}={\mathrm{NFD}}_{\theta }^{-1}\left(z;X_{l}\right)$. In ADANF, the latent variable *z* refers to the illumination-robust feature $F_{i}$. Similar to recent work [36], the latent variable *z* can be assumed to follow a Gaussian distribution $P_{z}\left(z\right)$. According to the change-of-variables theorem, the conditional distribution $P_{\mathrm{NFD}}\left(X_{gt}|X_{l},\theta \right)$ can be calculated as:

(7) $$\begin{array}{c} P_{\mathrm{NFD}}\left(X_{gt}|X_{l},\theta \right)=P_{z}\left(z\right)\left|\det\frac{\partial z}{\partial X_{gt}}\right|\\ \;\;\;\;\;\;\;\;\;\;=P_{z}\left({\mathrm{NFD}}_{\theta }\left(X_{gt};X_{l}\right)\right)\left|\det\frac{\partial {\mathrm{NFD}}_{\theta }\left(X_{gt};X_{l}\right)}{\partial X_{gt}}\right|. \end{array}$$

The normalizing flow decoder ${\mathrm{NFD}}_{\theta }$ is sequentially composed of *N* invertible layers $h^{n+1}={\mathrm{NFD}}_{\theta }^{n}\left(h^{n};{\mathrm{ADAE}}^{n}\left(X_{l}\right)\right)$, where ${\mathrm{NFD}}_{\theta }^{n}$ is the *n*-th layer, n=0,1,⋯,N−1, $h^{0}=X_{gt}$, and $h^{N}=z$. ${\mathrm{ADAE}}^{n}\left(X_{l}\right)$ is the latent image features from the adaptive dual aggregation encoder.

According to Equation (7), we can use the negative log-likelihood as a loss function L to optimize the parameters of the proposed ADANF. By using the chain rule, L is formulated as:

(8) $$\begin{array}{c} L=-\log P_{z}\left({\mathrm{NFD}}_{\theta }\left(X_{gt};X_{l}\right)\right)\\ \;\;\;\;\;\;\;\;\;-\sum_{n=0}^{N-1}\log\left|\det\frac{\partial {\mathrm{NFD}}_{\theta }^{n}\left(h^{n};{\mathrm{ADAE}}^{n}\left(X_{l}\right)\right)}{\partial h^{n}}\right|. \end{array}$$

Since $P_{z}\left(z\right)$ is assumed to follow a Gaussian distribution, $P_{z}\left({\mathrm{NFD}}_{\theta }\left(X_{gt};X_{l}\right)\right)$ can be calculated as:

(9) $$P_{z}\left({\mathrm{NFD}}_{\theta }\left(X_{gt};X_{l}\right)\right)=\frac{1}{\sqrt{2\pi }}\exp\left(\frac{-{\left(z-\mathrm{ADAE}\left(X_{l}\right)\right)}^{2}}{2}\right).$$

In the testing phase, low-light images are input to the adaptive dual aggregation encoder to obtain illumination-robust features, and then these features are input to the normalizing flow decoder through inverse mapping ${\mathrm{NFD}}_{\theta }^{-1}$ to generate normally exposed images.

## 4. Experiments

### 4.1. Datasets and Evaluation Metrics

Paired datasets. LOLv1 [21] is one of the most commonly used datasets in LLIE. This dataset is collected from real scenes and contains 500 pairs of low-light and normally exposed images under different lighting conditions. Among them, 485 pairs of images are used for training and 15 pairs of images are used for testing.

LOLv2 [22] contains two subsets, namely LOLv2-real and LOLv2-synthetic. LOLv2-real contains image pairs of different brightness in real scenes obtained by adjusting exposure time and ISO settings. These image pairs are intended to study illumination changes in real application scenarios. Specifically, LOLv2-real contains 689 image pairs for training and 100 image pairs for testing. LOLv2-synthetic synthesizes low-light images from RAW images by analyzing the lighting distribution of low-light images. It contains 1000 image pairs, of which 900 pairs are used for training and 100 pairs are used for testing.

Unpaired datasets. The DICM [55], LIME [3], MEF [56], NPE [57], and VV [46] (https://sites.google.com/site/vonikakis/datasets, accessed on 4 September 2023) datasets are real captured images and do not contain normally exposed images as reference images. Therefore, these datasets cannot be used for training. We tested the performance of the proposed ADANF on these several datasets.

Evaluation metrics. For paired datasets like LOL and LOL-v2, peak signal-to-noise ratio (PSNR) and structural similarity (SSIM) [58] are used as evaluation metrics. PSNR measures the peak signal-to-noise ratio between the original image and the enhanced image, while SSIM takes into account the structural and textural information of the image. In addition, learning perceptual image patch similarity (LPIPS) [59] is also used as an evaluation index, which uses deep features to measure the perceptual similarity of images. This indicator is learned through deep learning methods. Compared with PSNR and SSIM, LPIPS can more truly reflect the human eye’s perception of image quality.

For unpaired datasets such as DICM, LIME, MEF, NPE, and VV, direct evaluation using PSNR, SSIM, or LPIPS is not possible because there are no paired normally exposed images. We use the model parameters trained on LOLv2-synthetic to directly infer the enhanced image. In this case, the Natural Image Quality Evaluator (NIQE) is employed to evaluate the results. For PSNR and SSIM, the larger the value, the better the enhancement quality. For LPIPS and NIQE, the smaller the value, the better the enhancement quality.

### 4.2. Implement Details

In the proposed ADANF, the number of global–local adaptive aggregation modules in the adaptive dual aggregation encoder is 24, and the normalizing flow decoder has three levels with a squeeze layer and 12 flow steps at each level. The batch size on the LOLv1, LOLv2-real, and LOLv2-synthetic dataset is 8. We train the ADANF for 40,000 iterations using the Adam optimizer with the initial learning rate set to 0.0005 and multiplying the learning rate by 0.5 at 20,000, 30,000, 36,000, and 38,000 iterations. The input image size is set to 160×160. For unpaired data, we use the parameters trained on LOLv2-synthetic to perform inference and obtain results. All experiments are completed on a dual-card NVIDIA RTX 4090 server.

### 4.3. Comparisons with the State-of-the-Art Methods on Paired Datasets

In this section, to demonstrate the effectiveness of the proposed ADANF, we compare the ADANF with the state-of-the-art low-light image enhancement methods, such as LIME [3], Zero-DCE [47], RetinexNet [21], DRBN [60], KinD [43], KinD++ [61], EnlightenGAN [62], MIRNet [63], LLFlow [35], LLFormer [16].

The quantitative results of the proposed ADANF and comparison methods on the paired LOLv1, LOLv2-real, and LOLv2-synthetic datasets are reported in Table 1, Table 2 and Table 3, respectively. Low-light images often suffer from color distortion and low contrast, which make it difficult to extract effective features. Previous methods LIME [3], RetinexNet [21], and KinD [43] usually use classic structures when extracting low-light image features, which makes it difficult to effectively model their complex distribution when processing images under different low-light conditions, thus affecting performance. From Table 1, Table 2 and Table 3, we can see that our ADANF can be significantly improved under PSNR, SSIM, and LPIPS. It is worth noting that our ADANF has a greater improvement in the PSNR index, indicating that our proposed ADANF can obtain higher quality enhanced normally exposed images. Compared with the recent method LLFormer [16], the PSNR of our ADANF on the LOLv1, LOLv2-real, and LOLv2-synthetic datasets has increased by 0.91%, 1.81%, and 0.66%, respectively. This may be due to the fact that the adaptive dual aggregation encoder can effectively extract the global properties and local details from the low-light images. The proposed gated multi-scale information transmitting module can effectively transfer the latent features from the input image to the normalizing flow decoder so that the enhanced image has a more stable quality. The normalizing flow decoder can effectively model the distribution of normally exposed images to reconstruct high-quality images from illumination-robust features.

### 4.4. Comparisons with the State-of-the-Art Methods on Unpaired Datasets

In this section, we also conduct experiments on unpaired datasets. Due to the lack of reference images for comparison, we mainly used the NIQE to quantify the performance of each method and used the visual results for qualitative analysis. In terms of the NIQE indicator, the quantitative results for different datasets are shown in Table 4. The proposed ADANF shows better performance on the LIME, MEF, and VV datasets than other methods. On the DICM and NPE datasets, the proposed ADANF also has comparable performance.

### 4.5. Visualization

**Figure 3:**
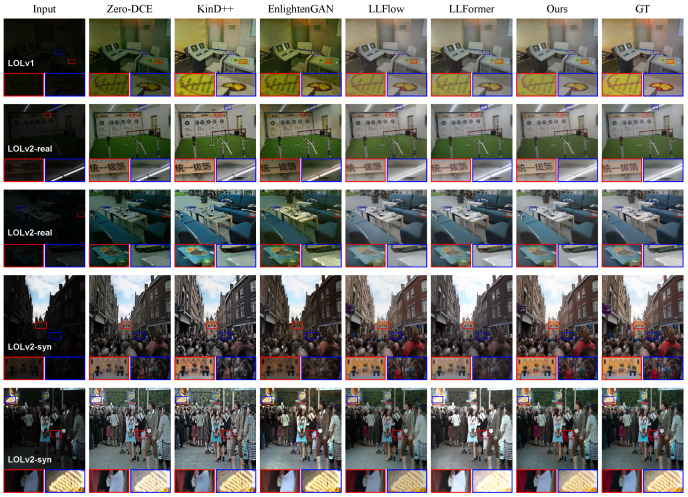
Some visualization results of the proposed ADANF and the recent state-of-the-art methods for the LOLv1, LOLv2-real, and LOLv2-synthetic datasets.

Visual results of different image enhancement methods on paired datasets. In order to verify that this method can generate better quality illumination-enhanced images, we compared some of the images generated by this method with the results generated by other low-light image enhancement algorithms. As shown in Figure 3, it can be seen that our ADANF can obtain a more realistic restoration effect. Compared with some methods, it has pictures with lower noise and more realistic colors. In addition, it has clearer details at the intersection of light and dark. These results can show that the module designed by this method is more complete and sufficient in extracting the features of the original image, promoting the final enhancement result to show more details in the transition area, thereby obtaining better enhancement results.

**Figure 4:**
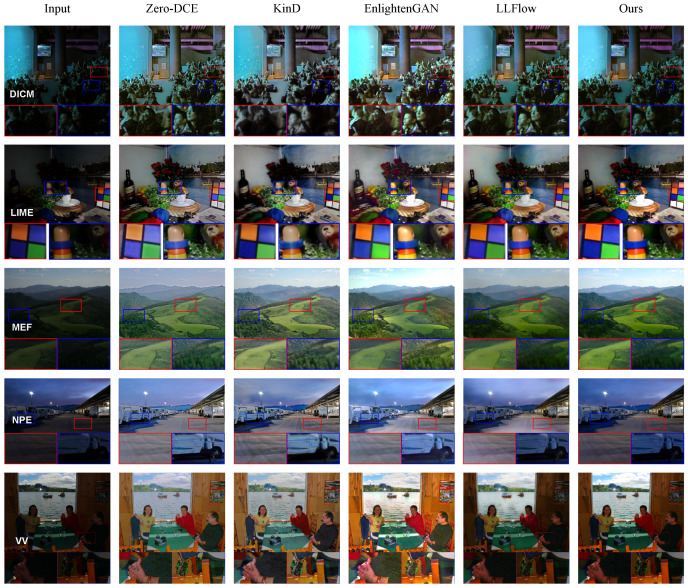
Some visualization results of the proposed ADANF and the recent state-of-the-art methods for the DICM, LIME, MEF, NPE, and VV datasets.

Visual results of different image enhancement methods on unpaired datasets. As can be seen from Figure 4, our method has better color performance in different scenarios. Compared with other methods, the image color obtained by this method is more realistic. It is neither too dark to see the details nor too bright to make the image color unrealistic. These visual results can show that the proposed method is effective not only in scenarios with paired datasets but also in complex scenarios with only unpaired datasets. Experimental results on multiple unpaired DICM, LIME, MEF, NPE, and VV datasets show that the proposed ADANF has good generalization ability.

### 4.6. Ablation Study

In order to verify the effectiveness of the different modules designed by this method, we conducted ablation experiments on the LOLv1 dataset to test the effects of the introduced adaptive dual aggregation encoder (ADAE) and gated multi-scale information transmitting module (GMITM). We replaced the ADAE in the proposed ADANF with multi-layer convolutions and removed the GMITM method as a baseline method. Then, the ADAE and GMITM modules are respectively added to the baseline method to conduct experiments. The experimental results are shown in Table 5. Compared with the baseline method, the introduction of the ADAE and GMITM alone can bring about improvements in the three evaluation indicators. The improvement brought by the introduction of the ADAE is that this module can fully utilize the potential features of the input image in both global and local aspects. The improvement obtained by further combining the GMITM and ADAE is because the image features extracted by ADAE are better transferred to the normalizing flow decoder to assist in image enhancement.

## 5. Conclusions

In this paper, we propose an adaptive dual aggregation network with normalizing flows for low-light image enhancement. First, an adaptive dual aggregation encoder is used to fully exploit the global properties and local details of the image to extract illumination-robust features. Next, after illumination-robust features are extracted, a reversible normalizing flow decoder is used to recover normally exposed images from these features. This step takes advantage of the inverse process capabilities of the normalizing flow decoder to reconstruct brighter, more detailed images from low-light images. Finally, a gated multi-scale information transmitting module is designed to introduce the multi-scale features of the adaptive dual aggregation encoder into the normalizing flow decoder. This step aims to further improve the quality of image enhancement by introducing multi-scale features. Extensive experiments on paired and unpaired datasets verify the effectiveness of the proposed ADANF. In the future, we will study lightweight low-light image enhancement networks to meet the needs of real-time low-light image processing applications.

## Figures and Tables

**Table 1:** Quantitative results of the proposed ADANF and the state-of-the-art methods for the LOLv1 datasets. ↑/↓ means that the larger/smaller the index value, the better/lower the quality. GFLOPs represents the Giga Floating Point Operations. Params represents the number of weight parameters.

Method	LOLv1				Complexity	
	PSNR↑	SSIM↑	LPIPS↓		GFLOPs	Params/M
LIME [3]	16.76	0.560	0.350		-	-
Zero-DCE [47]	14.86	0.562	0.335		-	0.33
RetinexNet [21]	16.77	0.462	0.474		587.47	0.84
DRBN [60]	19.86	0.834	0.155		48.61	5.27
KinD [43]	20.87	0.799	0.207		34.99	8.02
KinD++ [61]	21.30	0.823	0.175		-	9.63
EnlightenGAN [62]	17.48	0.652	0.322		61.01	114.35
MIRNet [63]	24.14	0.842	0.131		785	31.76
LLFlow [35]	25.13	0.872	0.117		-	37.68
LLFormer [16]	25.76	0.823	0.167		-	24.55
Diff-Retinex [64]	21.98	0.863	0.048		-	-
Transformer-GAN [65]	23.50	0.851	-		-	-
Restormer [66]	22.43	0.823	-		144.25	26.13
SNR-Aware [31]	26.72	0.851	0.152		26.35	4.01
ADANF (ours)	26.67	0.873	0.120		252.39	117.59

**Table 2:** Quantitative results of the proposed ADANF and the state-of-the-art methods for the LOLv2-real datasets. ↑/↓ means that the larger/smaller the index value, the better/lower the quality. GFLOPs represents the Giga Floating Point Operations. Params represents the number of weight parameters.

Method	LOLv2-Real				Complexity	
	PSNR↑	SSIM↑	LPIPS↓		GFLOPs	Params
LIME [3]	15.24	0.470	0.415		-	-
Zero-DCE [47]	18.06	0.580	0.313		-	0.33
RetinexNet [21]	18.37	0.723	0.365		587.47	0.84
DRBN [60]	20.13	0.830	0.147		48.61	5.27
KinD [43]	17.54	0.669	0.375		34.99	8.02
KinD++ [61]	19.09	0.817	0.180		-	9.63
EnlightenGAN [62]	18.64	0.677	0.309		61.01	114.35
MIRNet [63]	20.36	0.782	0.317		785	31.76
LLFlow [35]	26.20	0.888	0.137		-	37.68
LLFormer [16]	26.20	0.819	0.209		-	24.55
Restormer [66]	19.94	0.827	-		144.25	26.13
SNR-Aware [31]	27.21	0.871	0.157		26.35	4.01
ADANF(ours)	28.01	0.891	0.134		252.39	117.59

**Table 3:** Quantitative results of the proposed ADANF and the state-of-the-art methods for the LOLv2-synthetic datasets. ↑/↓ means that the larger/smaller the index value, the better/lower the quality. GFLOPs represents the Giga Floating Point Operations. Params represents the number of weight parameters.

Method	LOLv2-Synthetic				Complexity	
	PSNR↑	SSIM↑	LPIPS↓		FLOPs	Params
LIME [3]	16.88	0.776	0.675		-	-
RetinexNet [21]	17.13	0.798	0.754		587.47	0.84
DRBN [60]	23.22	0.927	-		48.61	5.27
KinD [43]	16.26	0.591	0.435		34.99	8.02
KinD++ [61]	-	-	-		-	9.63
EnlightenGAN [62]	16.57	0.734	-		61.01	114.35
MIRNet [63]	21.94	0.846	-		785	31.76
LLFlow [35]	24.81	0.919	0.067		-	37.68
LLFormer [16]	28.01	0.927	0.061		-	24.55
Restormer [66]	21.41	0.830	-		144.25	26.13
SNR-Aware [31]	27.79	0.941	0.054		26.35	4.01
ADANF(ours)	28.67	0.953	0.040		252.39	117.59

**Table 4:** Quantitative results of the proposed ADANF and the state-of-the-art methods for unpaired DICM, LIME, MEF, NPE, and VV datasets. The evaluation index is NIQE.

Methods	DICM	LIME	MEF	NPE	VV
Zero-DCE [47]	4.58	5.82	4.93	4.53	4.81
EnlightenGAN [62]	4.06	4.59	4.70	3.99	4.04
RetinexNet [21]	4.33	5.75	4.93	4.95	4.32
KinD [43]	3.95	4.42	4.45	3.92	3.72
KinD++ [61]	3.89	4.90	4.55	3.91	3.82
DCC-Net [67]	3.70	4.42	4.59	3.70	3.28
ADANF(ours)	3.90	3.78	3.59	4.24	3.14

**Table 5:** Ablation studies of the proposed ADANF on the LOLv1 dataset. ↑/↓ means that the larger/smaller the index value, the better/lower the quality.

ADAE	GMITM	PSNR↑	SSIM↑	LPIPS↓
		24.83	0.819	0.157
√		26.05	0.822	0.134
	√	25.91	0.845	0.126
√	√	26.67	0.873	0.120

## Data Availability

No new data were created or analyzed in this study. Data sharing is not applicable to this article.
